# Proper hepatic pedicle clamping during hepatectomy is associated with improved postoperative long-term prognosis in patients with AJCC stage IIIB hepatocellular carcinoma

**DOI:** 10.18632/oncotarget.8331

**Published:** 2016-03-24

**Authors:** Xiaoqiang Li, Shuang Liu, Hui Li, Lei Guo, Bo Zhang, Zhenhai Lin, Jubo Zhang, Qinghai Ye

**Affiliations:** ^1^ Liver Cancer Institute and Zhongshan Hospital, Fudan University, Shanghai 200032, P.R. China; ^2^ Key Laboratory of Carcinogenesis and Cancer Invasion, Ministry of Education, Shanghai 200032, P.R. China; ^3^ Department of Hepatic Surgery, Shanghai Cancer Center, Fudan University, Shanghai 200032, P.R. China; ^4^ Department of Infectious Diseases, Huashan Hospital, Fudan University, Shanghai 200040, P.R. China

**Keywords:** hepatocellular carcinoma, hepatic pedicle clamping, hepatectomy, tumour stage, prognosis

## Abstract

Intermittent hepatic pedicle clamping (HPC) is often performed during hepatectomy. Whether it affects the long-term prognosis of hepatocellular carcinoma (HCC) patients is still controversial. This study evaluated the impact of HPC in patients with different stages of HCC. The study included 1401 patients who underwent hepatectomy in the primary cohort with 129 AJCC stage IIIB HCC patients; there were 80 AJCC stage IIIB HCC patients in the validation cohort. In each cohort, patients were placed in the long-term HPC (LTHPC) group or the short-term HPC (STHPC) group based on the cut-off time of HPC estimated by the receiver-operating characteristic (ROC) curve. Although HPC did not show significant effects on the prognosis of stage I–IIIA HCC patients in the primary cohort, 1−, 3−, and 5-year overall survival (OS) and recurrence-free survival (RFS) rates of stage IIIB HCC patients who received LTHPC (HPC time > 12 minutes) were significantly higher than those with STHPC (HPC time ≤ 12 minutes or received no HPC), similar in the validation cohort. Multivariate analysis demonstrated HPC time was an independent protective factor for RFS and OS in stage IIIB HCC patients. Herein, we report that proper HPC improved the postoperative prognosis of stage IIIB HCC patients and served as an independent protective factor.

## INTRODUCTION

Primary liver cancer, 75–80% of which is hepatocellular carcinoma (HCC), is the fifth most common cancer and the second leading cause of cancer death in males worldwide [1]. Eastern Asia and sub-Saharan Africa are regions with the greatest incidence of HCC with a predominance of the male over the female gender (3/4:1) in the Asia-Pacific region, over 20 per 100,000 individuals [2]. Current guidelines do not recommend liver transplanting for HCC patients outside the Milan criteria because of the long time waiting for donor and absence of sufficient available evidence [3]. Percutaneous local ablative techniques are the choices in patients who are not candidates for surgery [4]. Liver resection is still the main curative treatment for HCC patients [5–7]. Unfortunately, even after curative hepatectomy, the postoperative recurrence-free survival (RFS) and overall survival (OS) rates were still unsatisfactory, with a 5-year recurrence rate of approximately 70% after hepatectomy [8]; prognosis is also poor in patients with advanced HCC (i.e., stage IIIB HCC). Therefore, how to improve the prognosis of HCC patients is still clinically concerning. Recently, several animal studies reported that hepatic ischemia–reperfusion (I/R) injury due to hepatic pedicle clamping (HPC) caused accelerated tumour growth and promoted metastases [9–12]. Whether this is the case in HCC patients is still controversial, but intermittent HPC is widely used to reduce blood loss during hepatectomy [13]. In 2005, Makino *et al.* reported the prognostic benefit of selective portal vein occlusion relative to total portal vein occlusion during hepatic resection for HCC patients [14]. Yang *et al.* also found that selective main portal vein occlusion could minimize the risk of recurrence after curative resection of HCC [15].

Given the conflicting data, the aim of this study was to explore whether the HPC could improve the recurrence-free and overall survival of HCC patients in a large, consecutive single institution cohort.

## RESULTS

### HCC patients with or without HPC did not show significant differences in prognosis

By the end of this study, 728 of 1401 patients had developed tumour recurrence (52.0%) (stage I: 45.7%, stage II: 56.4%; stage IIIa: 76.6%; stage IIIb: 58.9%) and 688 patients (49.1%) (stage I: 36.1%; stage II: 57.3%; stage IIIa: 76.6%; stage IIIb: 81.4%) had died. The 1−, 3−, and 5-year RFS and OS rates in patients without HPC during hepatectomy were relatively higher than those with HPC (76.3%, 54.8%, 47.2% versus 72.5%, 52.7%, 47%; 88.2%, 63.7%, 53.9% versus 82.1%, 61.4%, 53.8%), but with no significant differences. To find the best cut-off of HPC, rather than that simply defined as given HPC or not, we used the ROC curve (described in *Materials and Methods*). The best cut-off of HPC was 4 minutes for all patients of the primary cohort. Then, we redistributed the patients into the short-term hepatic pedicle clamping group (STHPC group; HPC time ≤ 4 min or without HPC; *n* = 993) and the long-term hepatic pedicle clamping group (LTHPC group; HPC time > 4 min; *n* = 408). The clinicopathological characteristics of the two groups are presented in Table S1. There were no significant differences in age, gender, HBsAg status, serum AFP concentration, multiplicity, tumour capsule, and Edmondson-Steiner grading. Lower rate of liver cirrhosis, bigger tumour size, and fewer stage I and stage II HCC patients were observed in the LTHPC group compared with the STHPC group (*P* = 0.005, *P* < 0.001, *P* = 0.002; Table S1). However, RFS and OS showed no significant differences between the STHPC and LTHPC groups (*P* > 0.05, data not shown).

### Prognosis improvement for stage IIIB HCC patients who received LTHPC in the primary cohort

**Figure 1:**
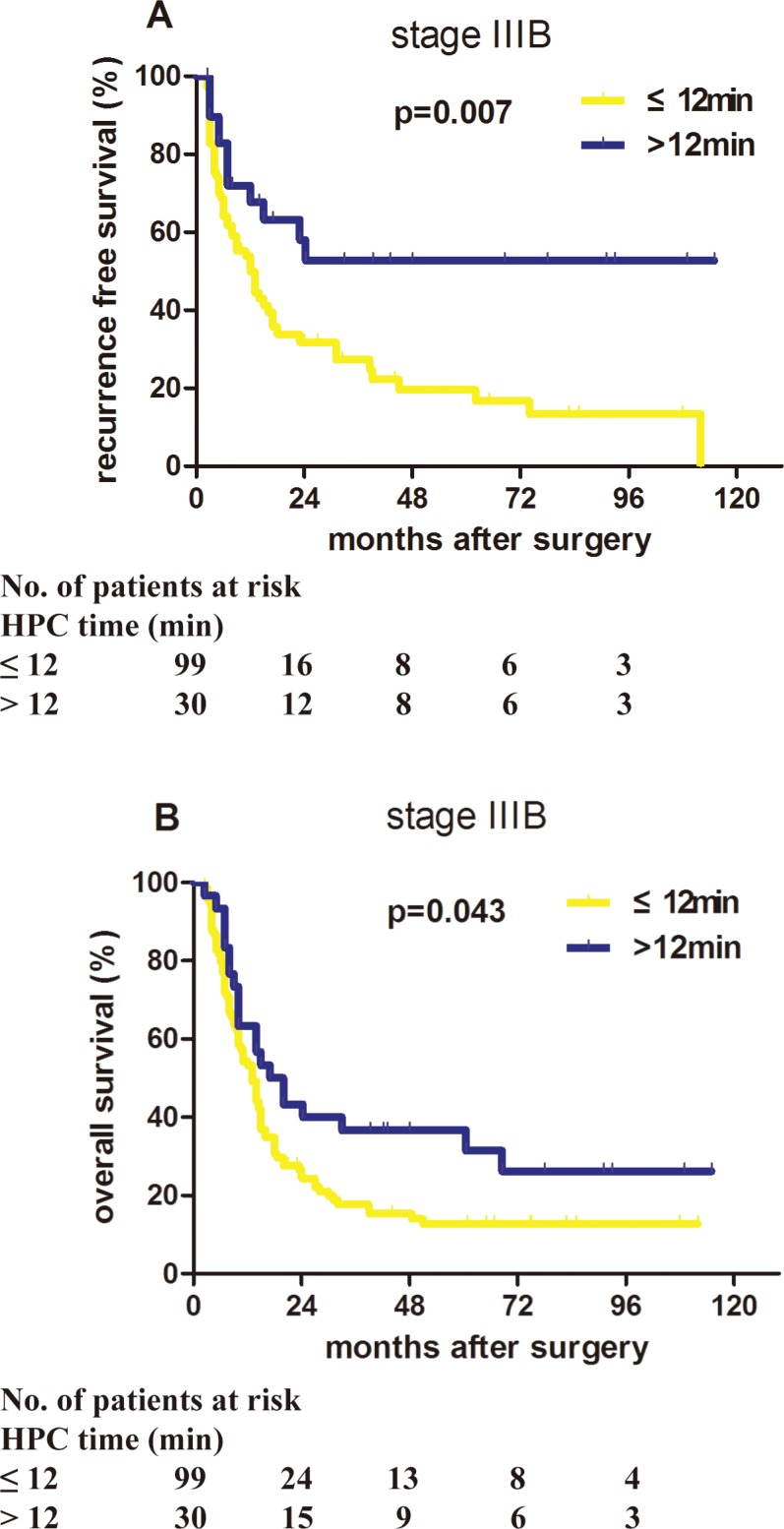
Proper HPC application benefited the prognosis of stage IIIB HCC patients in the primary cohort Patients with stage IIIB HCC in the primary cohort who received long-term HPC (HPC time > 12 minutes) had significantly better recurrence-free survival and overall survival than patients who received short-term HPC (HPC time ≤ 12 minutes). (**A**) *P* = 0.007. (**B**) *P* = 0.043.

As proposed, HCC patients with different clinical AJCC TNM stages might respond differently to HPC during surgery. ROC curve analysis was performed for patients with stage I, II, IIIA, and IIIB in the primary cohort, and the best cut-off times were 11, 13, 14, and 12 minutes, respectively. Whether HPC lasted longer than the cut-off time had no significant effects on RFS (*P* = 0.324, 0.219, 0.32) and OS (*P* = 0.344, 0.544, 0.085) of patients with stage I, II, and IIIA HCC (Figure S1). However, stage IIIB patients who received HPC for more than 12 minutes (LTHPC group, *n* = 30) had a better prognosis than those who received HPC for 12 minutes or less or those who did not receive HPC (STHPC group, *n* = 99; Figure 1). The clinicopathological characteristics of the two groups of stage IIIB patients are summarized in Table S2. There were no significant differences in these characteristics (Table S3), except that the proportion of patients with higher serum AFP concentration is larger in the STHPC group compared with the LTHPC group (*P* = 0.047).

In stage IIIB patients of the primary cohort with the median follow-up 14.0 months, the median RFS time of LTHPC group was significantly longer than that of the STHPC group (24.0 months vs. 13.0 months; *P* = 0.006), and 1−, 3−, and 5-year RFS rates of the LTHPC group were higher than those of the STHPC group (67.73% vs. 49.87%, 52.68% vs. 27.27%, 52.68% vs. 19.52%, respectively). Consistently, the median OS time was 34.28 months in the LTHPC group, which was longer than that (20.86 months) in the STHPC group (*P* = 0.046; Figure 1). The 1−, 3−, and 5-year OS rates of the LTHPC group were higher than those of the STHPC group (63.33% vs. 53.30%, 36.67% vs. 17.33%, 36.67% vs. 12.45%, respectively). Univariate analysis showed that the HPC time (*P* = 0.007), tumour capsule (*P* = 0.025), and tumour size (*P* = 0.029) were associated with RFS. Multivariate analysis using the Cox model demonstrated that the HPC time (*P* = 0.005, HR = 0.41, 95% CI = 0.220–0.765) and tumour capsule (*P* = 0.013, HR = 2.019, 95% CI = 1.157–3.523) were independent prognostic factors for RFS (Table 1). For OS, univariate analysis showed that the only significant protective factor was HPC time (*P* = 0.043; Table 1).

**Table 1:** Univariate and multivariate analyses of recurrence-free survival (RFS) and overall survival (OS) of stage IIIB HCC patients in the primary cohort (n = 129)

Characteristics	RFS			OS		
	*P* value	HR	95% CI	*P* value	HR	95% CI
Univariate analysis						
Sex: female vs. male	0.205	1.254	0.883–1.781	0.751	0.946	0.672–1.332
Age: > 50 vs. ≤ 50 years	0.331	0.721	0.491–1.054	0.426	0.854	0.579–1.259
HBsAg: positive vs. negative	0.756	1.056	0.592–1.886	0.811	1.076	0.589–1.964
AFP: > 20 vs. ≤ 20 ng/ml	0.846	1.060	0.590–1.905	0.372	1.268	0.753–2.134
Liver cirrhosis: no vs. yes	0.097	0.515	0.235–1.128	0.180	0.606	0.292–1.261
Tumour capsule: no vs. yes	0.025	1.888	1.084–3.289	0.500	1.096	0.840–1.431
Tumour size: > 5 vs. ≤ 5 cm	0.029	1.467	1.264–3.429	0.676	1.112	0.676–1.828
Tumour number: single vs. multiple	0.422	0.751	0.374–1.509	0.260	1.339	0.805–2.228
E-S Grading: I + II vs. III + IV	0.145	1.433	0.883–2.326	0.052	1.496	0.997–2.245
HPC time: > 12 vs. ≤ 12 min	0.007	0.428	0.230–0.797	0.043	0.609	0.376–0.985
Surgical procedures: I vs. II§	0.357	0.607	0.210–1.755	0.181	0.529	0.209–1.343
Operation time: ≤ 165 vs. > 165 min	0.856	1.037	0.703–1.529	0.634	0.908	0.611–1.350
Intraoperative blood loss: ≤ 1.1 vs. > 1.1 L	0.853	1.046	0.650–1.683	0.957	1.013	0.622–1.651
Intraoperative blood transfusion: no vs. yes	0.136	1.328	0.914–1.928	0.412	1.175	0.799–1.726
Multivariate analysis						
Tumour capsule: no vs. yes	0.013	2.019	1.157–3.523	NA		
Tumour size: > 5 vs. ≤ 5 cm	0.065	1.679	0.971–2.899	NA		
HPC time: >1 2 vs. ≤ 12 min	0.005	0.410	0.220–0.765	NA		

Abbreviations: *HCC* hepatocellular carcinoma, *HR* hazard ratio, *CI* confidence interval, *HBsAg* hepatitis B surface antigen, *AFP* α-fetoprotein, *HPC* hepatic pedicle clamping, *NA* not analysed.

§ Surgical procedures grade I: resection less than four liver segments. Surgical procedures grade II: resection of four or more liver segments.

### Verified prognosis improvement of stage IIIB patients who received LTHPC in a validation cohort

**Figure 2:**
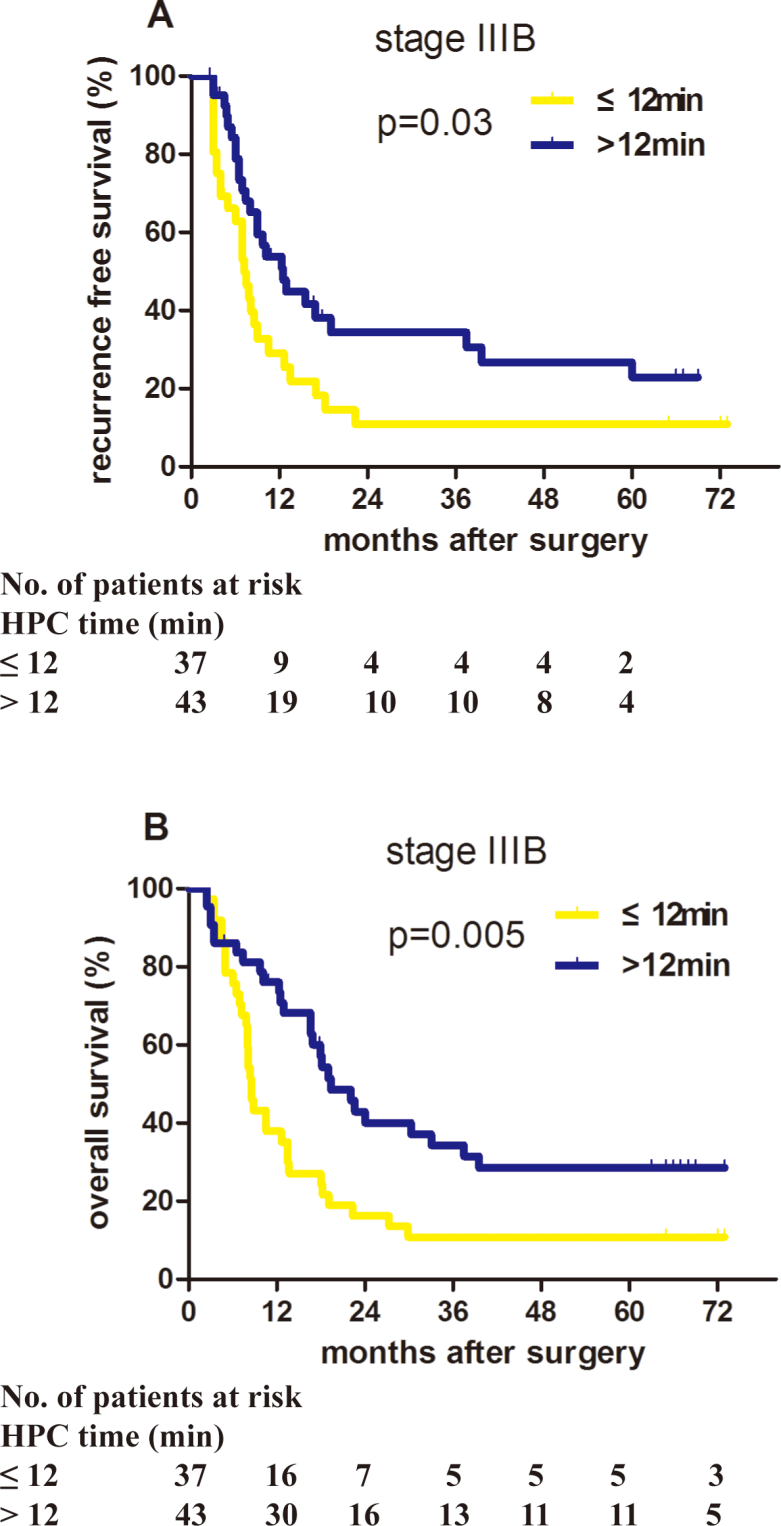
Proper HPC application benefited the prognosis of stage IIIB HCC patients in the validation cohort Patients with stage IIIB HCC in the validation cohort who received long-term HPC (HPC time > 12 min) had significantly better recurrence-free survival and overall survival than patients who received short-time HPC (HPC time ≤ 12 minutes). (**A**) *P* = 0.030. (**B**) *P* = 0.005.

To verify the importance of proper HPC during hepatectomy, another cohort of 80 patients with stage IIIB HCC was collected, with the median follow-up 12.6 months. These patients were also divided into the LTHPC group (HPC time > 12 minutes, *n* = 43) and the STHPC group (HPC time ≤ 12 minutes or without HPC, *n* = 37). The clinicopathological characteristics and surgical outcomes of these two groups in the validation cohort are summarized in Table S4 and Table S5, and no significant differences were observed between the two groups. The median RFS time of the LTHPC group was significantly longer than that of the STHPC group (12.5 months vs. 7.3 months, *P* = 0.030; Figure 2). The 1−, 3−, and 5-year RFS rates of the LTHPC group were higher than that of the STHPC group (53.79% vs. 29.08%, 34.33% vs. 10.90%, 22.89% vs.10.90%, respectively). The median OS time was 19.4 months in the LTHPC group, which was also longer than that of the STHPC group (8.5 months, *P* = 0.005; Figure 2). The 1-, 3-, and 5-year OS rates in the LTHPC group were higher than those in the STHPC group (76.06% vs. 37.84%, 34.29% vs. 10.81%, 28.58% vs.10.81%, respectively). Univariate analysis revealed that only HPC time was a protective factor affecting RFS (*P* = 0.030; Table 2). Using multivariate analysis for OS, HPC time is the only independent protective factor (*P* = 0.019, HR = 0.535, 95% CI = 0.317–0.904; Table 2).

**Table 2:** Univariate and multivariate analyses of recurrence-free survival (RFS) and overall survival (OS) of stage IIIB HCC patients in the validation cohort (n = 80)

Characteristics	RFS			OS		
	*P* value	HR	95% CI	*P* value	HR	95% CI
Univariate analysis						
Sex: female vs. male	0.521	1.466	0.455–4.723	0.875	1.098	0.343–3.509
Age: > 50 vs. ≤ 50 years	0.207	1.425	0.822–2.47	0.180	1.433	0.847–2.425
HBsAg: positive vs. negative	0.976	1.010	0.52–1.964	0.184	1.591	0.802–3.157
AFP: > 20 vs. ≤ 20 ng/ml	0.209	1.455	0.81–2.615	0.012	2.122	1.178–3.824
Liver cirrhosis: no vs. yes	0.284	0.604	0.24–1.519	0.298	0.615	0.246–1.538
Tumour capsule: no vs. yes	0.605	1.200	0.602–2.392	0.867	1.057	0.55–2.034
Tumour size: > 5 vs. ≤ 5 cm	0.764	1.101	0.586–2.069	0.032	2.06	1.064–3.987
Tumour number: single vs. multiple	0.851	1.08	0.486–2.397	0.811	1.102	0.498–2.435
E-S Grading: I + II vs. III + IV	0.582	0.858	0.498–1.479	0.816	1.062	0.639–1.765
HPC time: > 12 vs. ≤ 12 min	0.030	0.551	0.322–0.945	0.005	0.473	0.283–0.793
Surgical procedures: I vs. II§	0.235	0.723	0.423–1.235	0.847	1.057	0.602–1.856
Operation time: ≤ 165 vs. > 165 min	0.912	1.028	0.634–1.666	0.947	0.983	0.592–1.633
Intraoperative blood loss: ≤ 1.1 vs. > 1.1 L	0.590	0.831	0.423–1.631	0.422	1.382	0.627–3.045
Intraoperative blood transfusion: no vs. yes	0.689	0.898	0.531–1.520	0.748	1.096	0.625–1.923
Multivariate analysis						
AFP: > 20 vs. ≤ 20 ng/ml	NA			0.157	1.584	0.838–2.994
Tumour size: > 5 vs. ≤ 5 cm	NA			0.179	1.616	0.802–3.257
HPC time: > 12 vs. ≤ 12 min	NA			0.019	0.535	0.317–0.904

Abbreviations: *HCC* hepatocellular carcinoma, *HR* hazard ratio, *CI* confidence interval, *HBsAg* hepatitis B surface antigen, *AFP* α-fetoprotein, *HPC* hepatic pedicle clamping, *NA* not analysed.

§ Surgical procedures grade I: resection less than four liver segments. Surgical procedures grade II: resection of four or more liver segments.

## DISCUSSION

The best treatment for advanced HCC including stage IIIB remains controversial and can lead to high-risk intrahepatic recurrence and low survival rate [16]. Furthermore, portal vein tumour thrombus probably occurs in 10–40% of patients at the time of diagnosis and in 44% of patients with HCC at the time of death [17]. The median survival of untreated HCC patients with Vp4/3 was reported to be 2.7 months [16]. Another large-scale investigation of our institute revealed that the median survival of HCC patients with macroscopic portal vein tumour thrombus was 13.5 months [18]. In 2010, the Japan Society of Hepatology proposed that resection and transcatheter arterial chemoembolization (TACE) can be performed when there is portal invasion in HCC patients [19]. During hepatectomy, HPC was often applied to control blood loss to ensure a clear surgical field and to reduce the occurrence of postoperative liver failure [13]. In our study, there was no significant difference in surgical procedure, operation time, intraoperative blood loss, and blood transfusion between the LTHPC group and STHPC group. Thus, it cannot be argued that the possible negative effect was compensated by a decrease in operation time, intraoperative blood loss, and blood transfusion when HPC was used. It is common knowledge that HPC has long been used during elective hepatic resectional surgery to reduce operative blood loss and, in turn, decrease postoperative complications in hepatic tumour recurrence [20, 21]. In the present study using a primary cohort and a validation cohort, we concluded for the first time that proper HPC application would improve both RFS and OS of stage IIIB HCC patients. The cut-off time of HPC in the present study was 12 minutes. However, we observed no significant impact of HPC on prognosis of all subjects, consistent with the previous report.

Yamanaka *et al.* reported that during hepatectomy, especially in patients with portal invasion, tumour cells would dislodge into the portal vein, and thus into the remnant liver, which would directly cause recurrence [22]. Similar results were also reported in pancreatic cancer, [23] lung cancer, [24] and colorectal cancer, [25] indicating that compression of tumours during operation would increase circulating tumor cell (CTC) numbers. It is predictable that CTC dissemination requires patency blood flow, which is blocked when HPC is adopted during hepatectomy. This may explain why HPC functioned as a protective factor in stage IIIB HCC patients.

However, I/R injury caused by HPC might have a negative impact on the prognosis. Several animal studies did demonstrate that I/R injury caused by HPC would promote tumour growth and metastasis in both HCC and colorectal liver metastasis models [9, 26]. Results of clinical studies, however, were more complicated. Evidence presented by Yang *et al.* showed that selective main portal vein occlusion could minimise the risk of recurrence after curative resection of HCC [15]. Recently, a case-matched study involving colorectal liver metastasis patients figured that the 5-year recurrence-free rate of HPC patients was significantly higher than that of patients who did not receive HPC [27]. However, it was rather remarkable that another study conducted by Xia *et al.* described no significant results between HCC patients undergoing HPC or not during hepatectomy [28]. Other studies also observed no significant impact of HPC on the prognosis of patients with colorectal liver metastasis [29–33]. In the present study, no significant impact of HPC on the prognosis of all subjects was observed, consistent with previous reports by Xia *et al.* Interestingly, Makino *et al.* [28]. reported that selective portal vein occlusion, compared with total portal vein occlusion, during hepatic resection allowed better prognosis for HCC patients [14]. In this study, better prognosis was only shown in the selective portal vein occlusion (SPVO) group, in which blood flow was blocked in the tumour-bearing side with minimal I/R injury of the remnant liver. Moreover, the SPVO group also had reduced impact of tumour cell dissemination. Because there was no consideration of CTC dissemination, this might explain the high rate of metastasis and recurrence in HCC animal models caused by HPC-induced I/R injury. Only when CTC dissemination and I/R injury were taken into consideration could we exactly evaluate the role of HPC in HCC prognosis. Another study reported by Tanaka *et al.* might provide great evidence for the interaction of CTC dissemination and I/R injury. They showed that HPC during hepatectomy significantly reduced postoperative metastasis to extrahepatic sites, whereas no significance was observed for intrahepatic recurrence [34].

This study has several inherent limitation that should be addressed. It's a retrospective study to discuss the effects of HPC on patients' outcomes after hepatic resection. The findings may limited by selection bias and confounding factors, which could be minimize a prospective randomized controlled study. It may lead to selection bias because of the use of HPC mainly depending on the individual surgeons' preference during operation. The two cohorts including primary cohort and validation cohort may reduce the selection bias and the confounding factors.

To our knowledge, we were the first to conclude that although HPC had no impact on the prognosis of all stages of I–IIIA HCC patients, consistent with the previous results reported by Xia *et al.*, [28] proper application of HPC was a protective factor for stage IIIB HCC patients who were suffering from macroscopic vascular invasion. Future studies should be conducted to determine if HPC during hepatectomy will reduce tumour cell dissemination, to clarify the interaction between tumour cell dissemination and I/R injury, and to confirm whether selective main portal vein occlusion [15] or portal vein clamping [14] would be more positive for the prognosis of stage IIIB HCC patients. In the present study, we found that proper HPC did improve the postoperative prognosis of stage IIIB HCC patients. We proposed that if the parenchymal liver resection time was preoperatively evaluated over 12 minutes, then proper HPC should be performed during hepatectomy for improvement in prognosis.

## MATERIALS AND METHODS

### Patients

**Figure 3:**
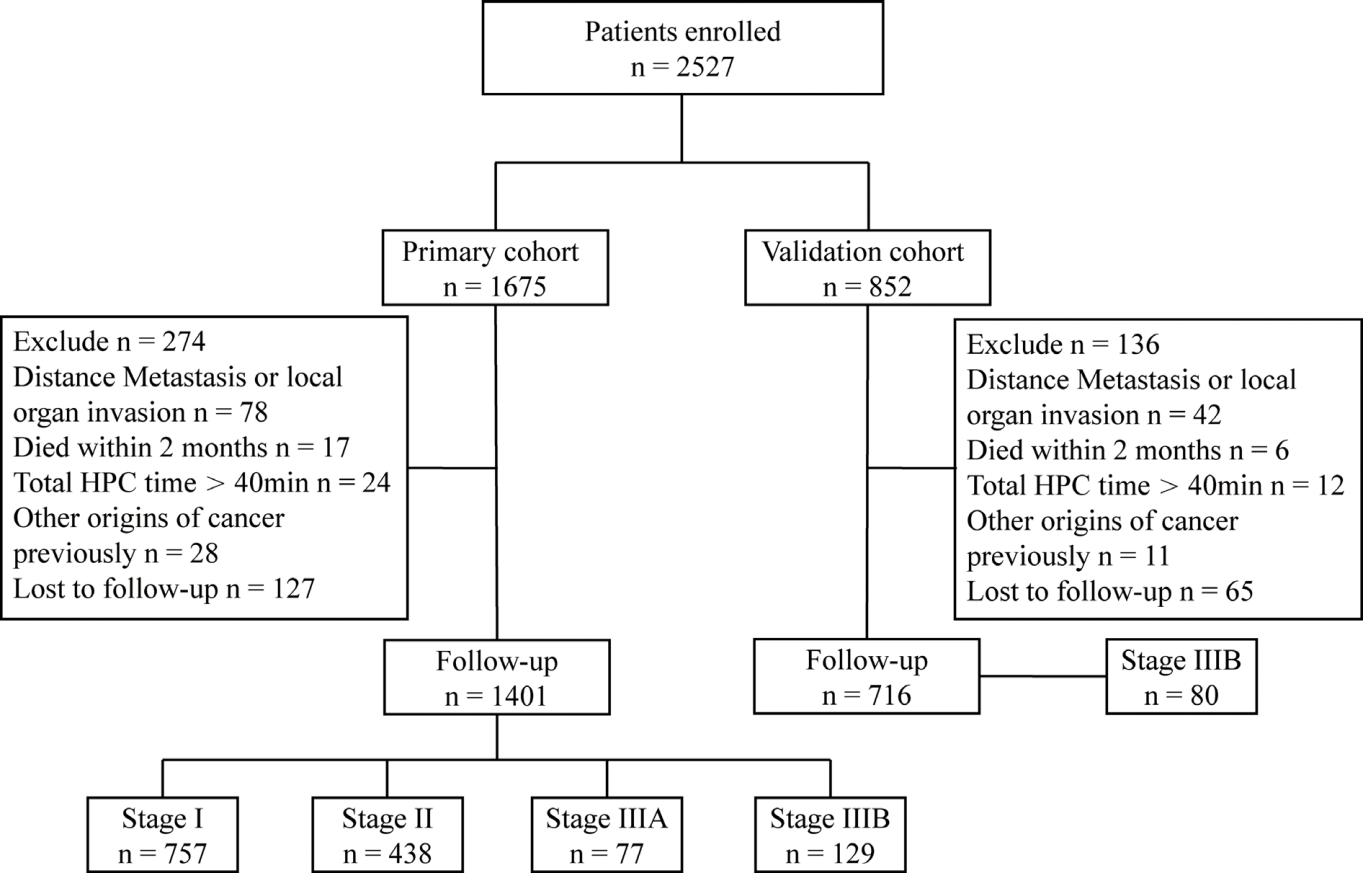
Study design From January 2003 to December 2006, 1401 patients undergoing tumour resection in Zhongshan Hospital were retrospectively included in the primary cohort, which consisted of 757 stage I, 438 stage II, 77 stage IIIA, and 129 stage IIIB HCC patients. The primary cohort was used to determine the baseline HPC time and investigate the impact of HPC time on prognosis. Another 80 stage IIIB HCC patients also in Zhongshan Hospital from January 2007 to December 2007 were included in the validation cohort. This cohort was used to verify the improvement of the prognosis with proper HPC in stage IIIB HCC patients.

Tumour stages were determined by the 2010 American Joint Committee on Cancer (AJCC) 7th TNM staging system [35]. From January 2003 to December 2006, 2527 consecutive patients underwent liver resection by the same medical center. The status of disease and resectability were assessed by contrast-enhanced magnetic imaging, ultrasonography, and computed tomography. 1401 HCC patients with stage I/II/IIIA/IIIB HCC undergoing tumour resection in Zhongshan Hospital were retrospectively included in the primary cohort of the present study; 80 stage IIIB HCC patients underwent curative resection from January 2007 to December 2007 were included in the validation cohort (Figure 3). This study was approved by the Research Ethics Committee of Zhongshan Hospital, and informed consent was obtained from all subjects.

The inclusion criteria were as follows: (1) HCC diagnosed pathologically after resection and (2) patients underwent curative resection for HCC, defined as complete macroscopic removal of the tumour and cancerous thrombus. The exclusion criteria were as follows: (1) patients with distant metastasis or local organ invasion; (2) patients with other origins of cancer previously; (3) patients who died within 30 days after surgery; (4) total HPC time exceeded 40 minutes or HPC was used more than twice during the operation; (5) patients received other treatment prior to liver resection; and (6) patients were lost at the beginning of the follow-up.

All patients were monitored by serum alpha-fetoprotein (AFP), abdomen ultrasonography, and chest X-ray every 1 to 6 months and magnetic resonance imaging (MRI) every 6 to 12 months according to the postoperative time. For patients with test results suggestive of recurrence, computed tomography and/or magnetic resonance imaging were used to verify whether recurrence had occurred. The diagnosis of recurrence was based on the combination of imaging and clinical evaluations.

Patients lost to follow-up were censored at their last encounter. Mortality was defined as death within the first 30 postoperative days, and these patients were excluded as described in the exclusion criteria.

### Clinicopathological factors

Clinicopathological factors in this study were selected for their potential relation to the prognosis on the basis of the previous studies [36, 37], including age (50 years or younger or older than 50 years), gender (male or female), HBsAg status (positive or negative), liver cirrhosis (yes or no), serum AFP concentration (≤ 20 or > 20 ng/mL), tumour size (≤ 5 or > 5 cm), number of tumour nodules (solitary or multiple), tumour capsule (positive or negative), and differentiation of tumour cells (Edmondson's classification I/II or III/IV) [38]. The characteristics regarding how surgical outcomes may affect the prognosis were also selected based on the previous study, including surgical procedure (I, ≤ 3 segments; II, > 3 segments), and based on Couinaud's nomenclature [39], operation time, intraoperative blood loss, and intraoperative blood transfusion (yes or no). For the laboratory parameters, the cutoff values were the upper limit of the normal values in our hospital.

### Treatment

Liver resection included nonanatomical resection, subsegmentectomy, segmentectomy, hemihepatectomy, and trisecmentectomy. A major hepatectomy is defined as resection of four or more Couinaud's segments. The extent of hepatectomy was evaluated according to the extent of disease progression, liver function, and general status of patients. HPC (i.e., Pringle manoeuver), if used, was achieved by tightening 4-mm tape around the portal triad. Intermittent HPC consisted of cycles of 15–20 min clamping followed by 5 minutes unclamping. The use of HPC was based on the individual preference of surgeons during operation. The time of HPC was recorded by the nurse.

### Statistics

The RFS time was defined as the interval between the operation and the date of diagnosis of the first recurrence. The OS time was defined as the interval between the operation and death. The optimal cut-off time of HPC was assessed by the receiver-operator characteristic (ROC) curve and estimated the area under the curve (using the calculator for ROC curves available from Johns Hopkins University, Baltimore, MD, USA [www.jrocfit.org, accessed 30 April 2008]). Each point on the ROC curve represents a sensitivity/specificity pair corresponding to a particular decision threshold. A best test shows the ROC curve that passes through the upper left-hand corner (100% sensitivity, 100% specificity) [40–42]. Therefore the larger area under the ROC curve means the higher overall accuracy of the test. The values yielding maximum sums from the ROC curves got the best sensitivity and specificity. Associations between HPC time and patient characteristics were analyzed using the χ^2^ test (or Fisher's exact test) for two categorical variables. Survival curves were calculated by the Kaplan-Meier method and compared by log-rank test. Multivariate analysis was performed using Cox proportional hazards model. Stratified log-rank tests were used to test the effect of HPC whilst controlling for the effect of another variable. Variables with *P* < 0.05 on univariate analysis were further evaluated by multivariate analysis.

## SUPPLEMENTARY MATERIALS TABLES AND FIGURE


