# Supplementary material for: Proper hepatic pedicle clamping during hepatectomy is associated with improved postoperative long-term prognosis in patients with AJCC stage IIIB hepatocellular carcinoma
Source: Oncotarget. 2016 Mar 24;7(17):24623–32. doi: 10.18632/oncotarget.8331 (PMC5029728; doi:10.18632/oncotarget.8331)
Supplement: Supplementary file 1 [file oncotarget-07-24623-s001.pdf]

Proper hepatic pedicle clamping during hepatectomy is associated with improved postoperative long-term prognosis in patients with AJCC stage IIIB hepatocellular carcinoma

Supplementary Materials

Supplementary Table S1: Comparison of characteristics between all HCC patients with different HPC status in primary cohort (*n* = 1401)

Characteristics	HPC > 4 min		HPC ≤ 4 min or without		*P* value
	No. of patients	%	No. of patients	%	
Age					0.572
≤ 50	190	46.6	446	44.9	
> 50	218	53.4	547	55.1	
Sex					0.253
Male	356	87.3	843	84.9	
Female	52	12.7	150	15.1	
HBsAg					0.092
Positive	346	84.8	875	88.1	
Negative	62	15.2	118	11.9	
Liver cirrhosis					0.005
Yes	336	82.4	871	87.7	
No	73	17.6	121	12.3	
AFP					0.363
> 20 ng/ml	277	67.9	649	65.4	
≤ 20 ng/ml	131	32.1	344	34.6	
Tumour size					< 0.001
> 5 cm	244	59.8	370	37.3	
≤ 5 cm	164	40.2	623	62.7	
Multiplicity					0.967
Multiple	58	14.2	142	14.3	
Solitary	350	85.8	851	85.7	
Tumour capsule					0.108
Negative	212	52.0	469	47.2	
Positive	196	48.0	524	52.8	
Macrovascular invasion					0.055
Positive	47	11.5	82	9.0	
Negative	361	88.5	911	91.0	
Edmondson-Steiner Grading					0.376
I + II	105	25.7	234	23.6	
III + IV	302	74.3	759	76.4	
TNM stage					0.002
I + II	329	80.6	866	87.2	
IIIA + IIIB	79	19.4	127	12.8	

HCC hepatocellular carcinoma, *HR* hazard ratio, *CI* confidence interval, *HBsAg* hepatitis B surface antigen, AFP α-fetoprotein, *HPC* hepatic pedicle clamping.

Supplementary Table S2: Comparison of characteristics between stage IIIB HCC patients with different HPC status in primary cohort (n = 129)

Characteristics	HPC > 12 min		HPC ≤ 12 min or without		p value
	No. of patients	%	No. of patients	%	
Age					0.992
≤ 50	17	56.7	56	56.6	
> 50	13	43.3	43	43.4	
Sex					0.068
Male	25	83.3	93	93.9	
Female	5	16.7	6	6.1	
HBsAg					0.762§
Positive	27	90.0	86	86.9	
Negative	3	10.0	13	13.1	
Liver cirrhosis					0.433§
Positive	27	90.0	93	93.9	
Negative	3	10.0	6	6.1	
AFP					0.047
> 20 ng/ml	21	70.0	85	85.9	
≤ 20 ng/ml	9	30.0	14	14.1	
Tumor size					0.823
> 5 cm	24	80.0	81	81.8	
≤ 5 cm	6	20.0	18	18.2	
Multiplicity					0.158§
Multiple	2	6.7	18	18.2	
Solitary	28	93.3	81	81.8	
Tumor capsule					0.352
Negative	24	80.0	86	86.9	
Positive	6	20.0	13	13.1	
Edmondson-Steiner Grading					0.513
I + II	22	73.3	67	67.7	
III + IV	8	26.7	33	32.3	

§Twenty-five percent of all the cells have expected count less than 5; Fisher's exact test.
HCC hepatocellular carcinoma, *HR* hazard ratio, *CI* confidence interval, *HBsAg* hepatitis B surface antigen, *AFP* α-fetoprotein, *HPC* hepatic pedicle clamping.

Supplementary Table S3: Comparison of characteristics about surgical outcomes (Operation-related factors) between stage IIIB HCC patients with different HPC status in primary cohort (*n* = 129)

Characteristics	HPC > 12 min	HPC ≤ 12 min or without	*P* value
Surgical procedures§:			
I	26	74	0.260
II	9	20	
Operation time(min)	185.00 ± 31.07	174.65 ± 50.93	0.294
Intraoperative blood loss (mL)	776.67 ± 592.15	758.98 ± 584.05	0.885
Intraoperative blood loss (L)			
≤ 1.1	25	80	0.756
> 1.1	5	19	
Intraoperative blood transfusion			
Performed	11	45	0.395
Not performed	19	54	
Intraoperative blood transfusion(mL)	440.00 ± 750.91	483.33 ± 707.07	0.772

§Surgical procedures grade I: resection less than four liver segments. Surgical procedures grade II: resection of four or more liver segments.

Supplementary Table S4: Comparison of characteristics between stage IIIB HCC patients with different HPC status in validation cohort (n = 80)

Characteristics	HPC > 12 min		HPC ≤ 12 min or without		*P* value
	No. of patients	%	No. of patients	%	
Age					0.260
≤ 50	21	48.8	13	35.1	
> 50	22	51.2	24	64.9	
Sex					1.000§
Male	41	95.3	36	97.3	
Female	2	4.7	1	2.7	
HBsAg					0.150§
Positive	32	74.4	33	89.2	
Negative	11	25.6	4	10.8	
Liver cirrhosis					0.366§
Positive	39	90.7	36	97.3	
Negative	4	9.3	1	2.7	
AFP					0.085
> 20 ng/ml	26	60.5	29	78.4	
≤ 20 ng/ml	17	39.5	8	21.6	
Tumour size					0.861
> 5 cm	33	76.7	29	78.4	
≤ 5 cm	10	23.3	8	21.6	
Multiplicity					0.745§
Multiple	6	14.0	4	10.8	
Solitary	37	86.0	33	89.2	
Tumour capsule					0.590
Negative	34	79.1	31	83.8	
Positive	9	20.9	6	16.2	
Edmondson-Steiner Grading					0.770
I + II	23	53.5	21	56.8	
III + IV	20	46.5	16	43.2	

§Twenty-five percent of all the cells have expected counts less than 5; Fisher's exact test.

HCC hepatocellular carcinoma, *HR* hazard ratio, *CI* confidence interval, *HBsAg* hepatitis B surface antigen, *AFP* α-fetoprotein, *HPC* hepatic pedicle clamping.

Supplementary Table S5: Comparison of characteristics of surgical outcomes (operation-related factors) between stage IIIB HCC patients with different HPC status in validation cohort (*n* = 80)

Characteristics	HPC > 12 min	HPC ≤ 12 min or without	*P* value
Surgical procedures:			
I	29	30	0.167
II	14	7	
Operation time (min)	178.37 ± 51.97	160.41 ± 55.77	0.140
Intraoperative blood loss (mL)	653.49 ± 556.93	575.68 ± 571.62	0.540
Intraoperative blood loss (L)			
≤ 1.1	35	34	0.174
> 1.1	8	3	
Intraoperative blood transfusion			
Performed	12	11	0.395
Not performed	31	26	
Intraoperative blood transfusion (mL)	262.79 ± 520.08	294.59 ± 571.52	0.857

§Surgical procedures grade I: resection less than four liver segments. Surgical procedures grade II: resection of four or more liver segments.

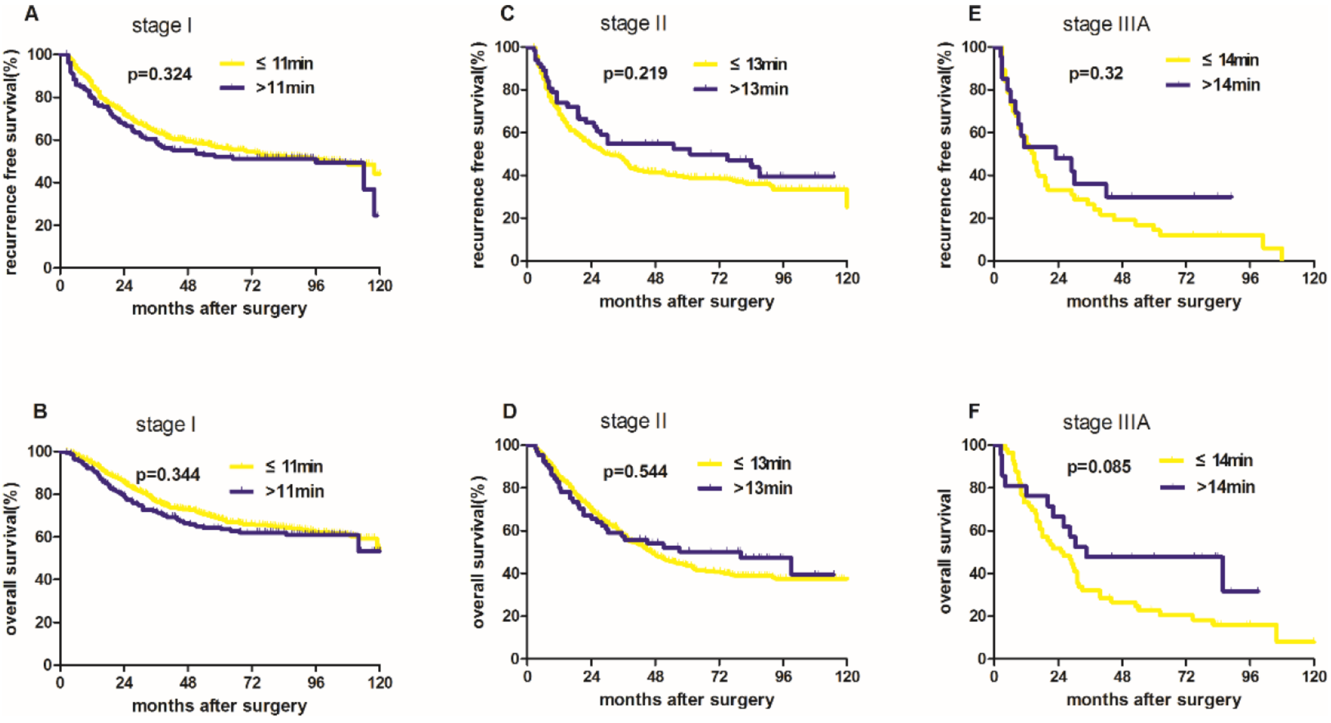

Supplementary Figure S1: Effects of HPC application on prognosis of patients with stage I, II, and IIIA HCC in the primary cohort. There were no significant differences in recurrence-free survival and overall survival in the LTHPC and STHPC groups.
